# Supplementary figures and images for: Mating disrupts morning anticipation in Drosophila melanogaster females
Source: PLoS Genet. 2022 Dec 22;18(12):e1010258. doi: 10.1371/journal.pgen.1010258 (PMC9779042; doi:10.1371/journal.pgen.1010258)

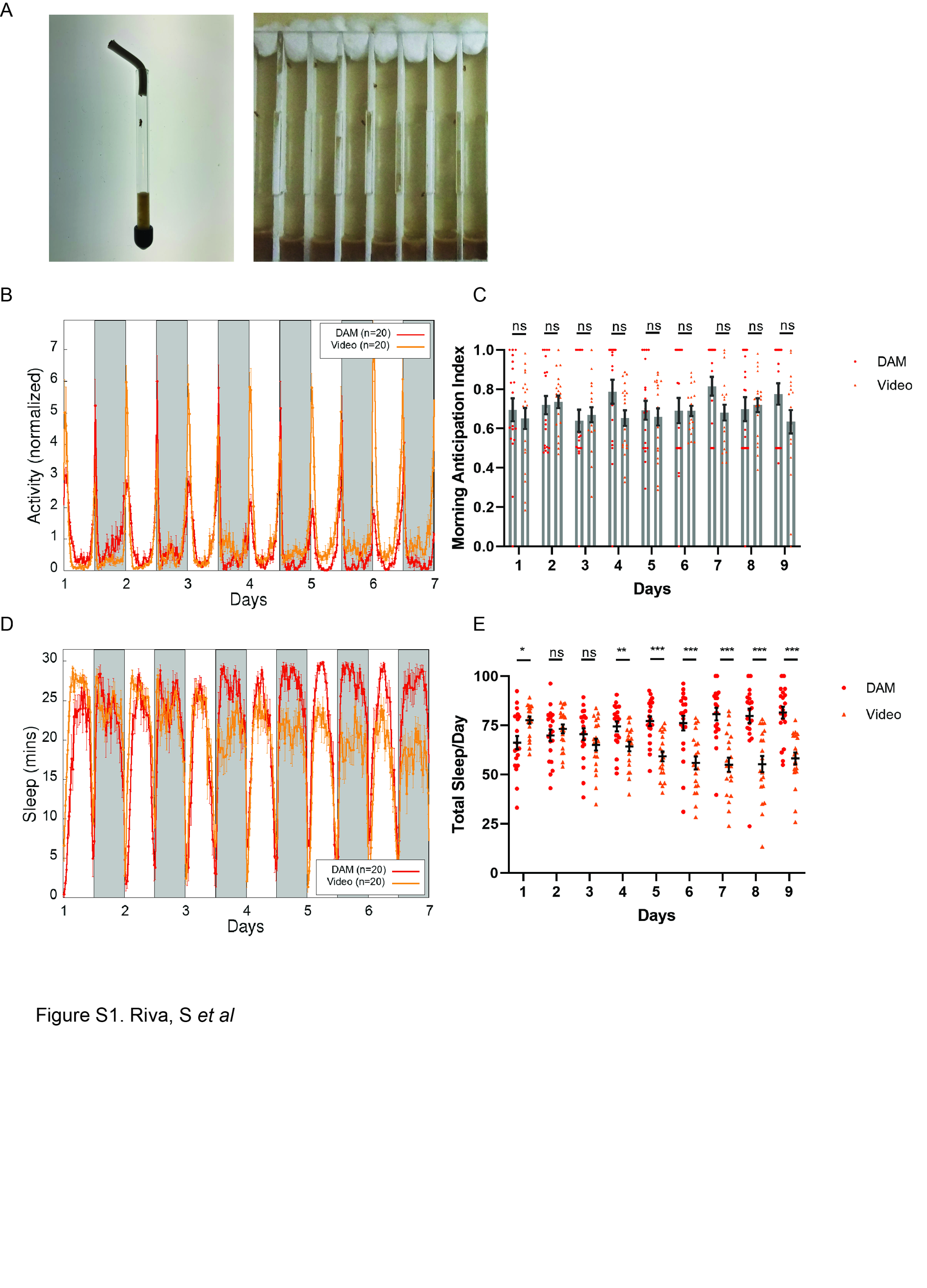

Supplement: S1 Fig — (A) Left: Tube where a fly is housed in the DAM system. Right: Set of chambers where flies are housed in our video tracking system. (B) Comparison between group average locomotor activity of male CantonS flies in LD, obtained using both systems. (C) Comparison between Morning Anticipation Index of male CantonS flies, obtained using DAM (red) and Video sytems (orange). Each dot corresponds to the index calculated for a single fly. Statistical analysis: Scheirer–Ray–Hare test. Post hoc tests Wilcoxon rank tests for every day, corrected for multiple comparisons (Benjamini-Hochberg). (D) Comparison between the times spent sleeping (in 30 minutes bins) of male CantonS flies obtained using both systems under LD conditions. (E) Comparison between total sleep of male CantonS flies obtained using DAM (red) and video (orange) systems under LD conditions. Each dot corresponds to the percentage of sleep per day calculated for a single fly. Statistical analysis: Scheirer–Ray–Hare test, as Post hoc tests we have applied Wilcoxon rank tests for every day, corrected for multiple comparisons (Benjamini-Hochberg). Dots represent independent flies, the mean and SEM are shown. *p< 0.05, **p< 0.01 ***p < 0.001. ns, not significant. (TIF) [file pgen.1010258.s001.tif]

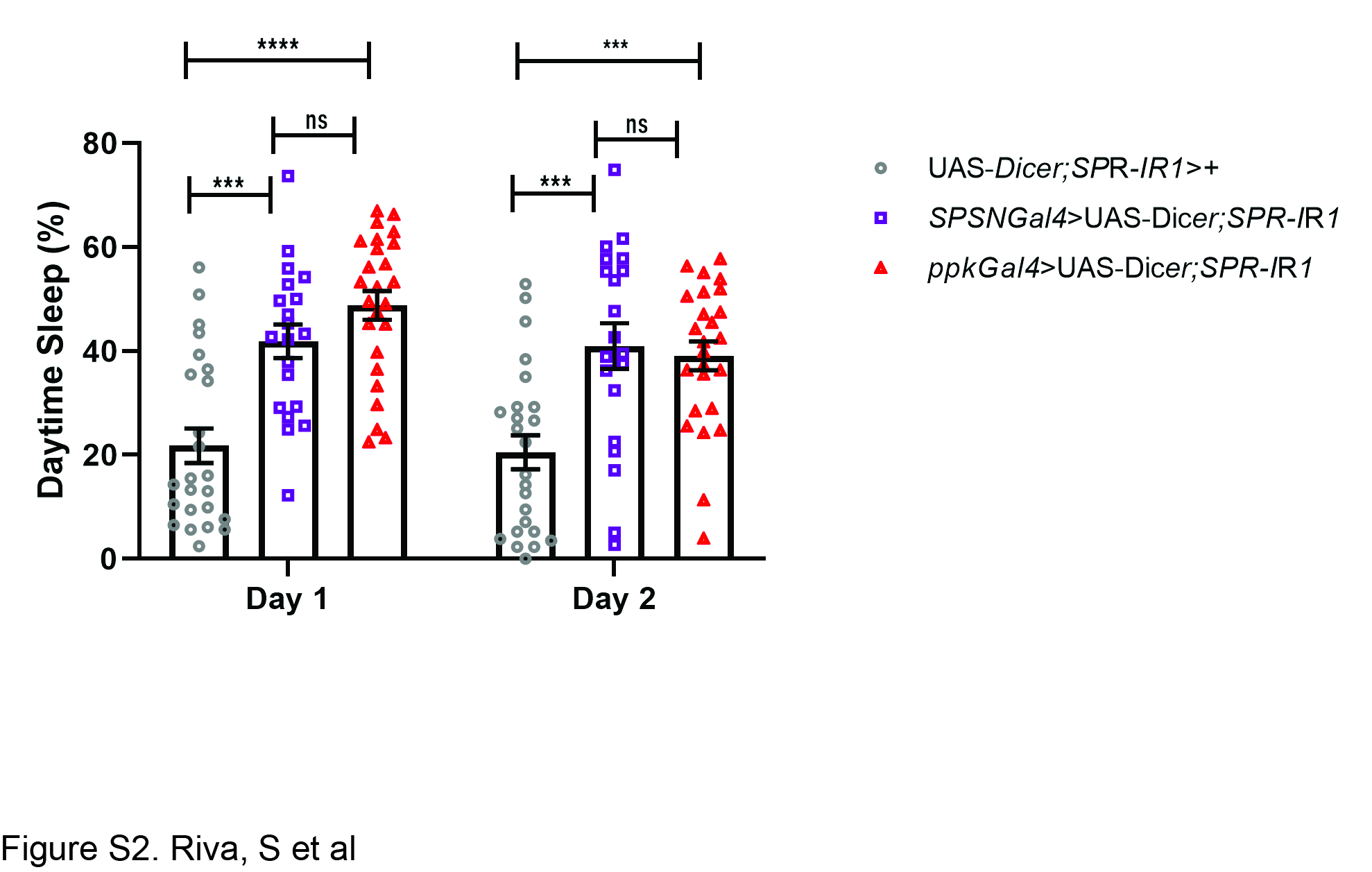

Supplement: S2 Fig — Total daytime sleep (ZT0-12) of the first and second day after male removal of mated females of the indicated genotypes. ****p < 0.0001; ***p ≤ 0.001; ns, not significant. (TIF) [file pgen.1010258.s002.tif]

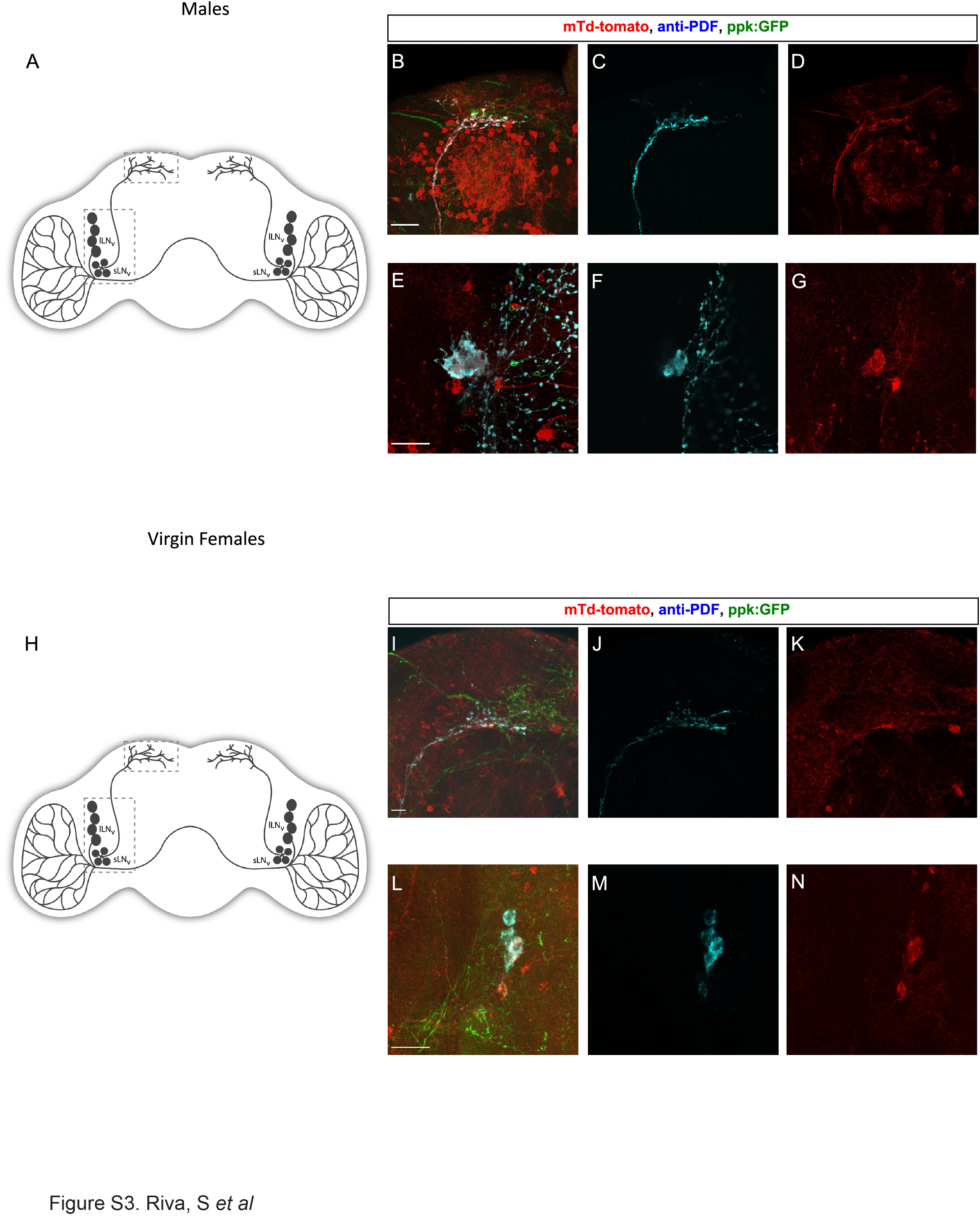

Supplement: S3 Fig — Trans-synaptic labeling using trans-Tango. (A) Schematic diagram of a Drosophila brain. (B) Higher magnification of the dorsal region of ppk-Gal4>UAS-myr-GFP, QUAS-mtdTom; trans-Tango male brain. (C, D) Single focal plane of the image shown in B displaying the overlap of the PDF staining (cyan) with the postsynaptic ppk+ partners (red). E, Higher magnification of the accessory medulla region of ppk-Gal4>UAS-myr-GFP, QUAS-mtdTom; trans-Tango male brain. (F, G) Single focal plane of the image shown in E to exhibit the overlap of the PDF stain with the postsynaptic ppk partners (red). (H) Schematic diagram of a fly brain. (I) Higher magnification of the dorsal region of ppk-Gal4>UAS-myr-GFP, QUAS-mtdTom; trans-Tango virgin female brain. (J, K) Single focal plane of the image shown in I displaying the overlap of the PDF stain (cyan) with the postsynaptic ppk partners (red). (L) Higher magnification of the accessory medulla region of ppk-Gal4>UAS-myr-GFP, QUAS-mtdTom; trans-Tango virgin brain. (M, N) Single focal plane of the image shown in L to show the overlap of the PDF stain with the postsynaptic ppk partners (red). (TIF) [file pgen.1010258.s003.tif]
